# Childhood Blood Lead Levels in Children Aged <5 Years — United States, 2009–2014

**DOI:** 10.15585/mmwr.ss6603a1

**Published:** 2017-01-20

**Authors:** Jaime Raymond, Mary Jean Brown

**Affiliations:** 1 Division of Emergency and Environmental Health Services, National Center for Environmental Health

## Preface

**FIGURE 1:**
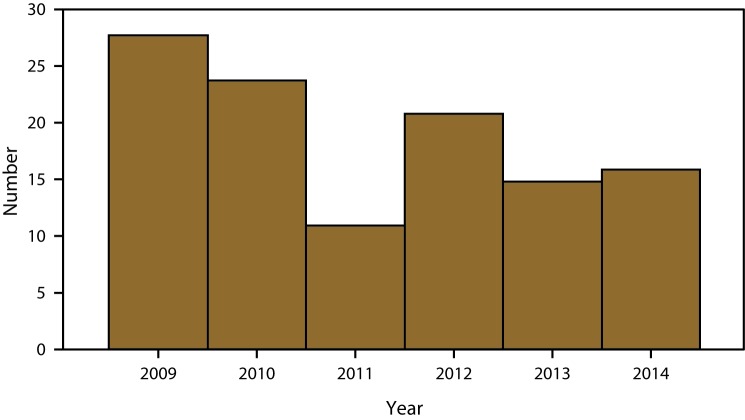
Number of children aged <5 years with newly confirmed blood lead levels ≥70 *μ*gL — Childhood Blood Lead Surveillance System, United States, 2009–2014* *For 2009, a total of 38 jurisdictions reported data to CDC (Alabama, Arizona, California, Connecticut, Delaware, District of Columbia, Florida, Georgia, Illinois, Indiana, Iowa, Kansas, Kentucky, Louisiana, Maine, Maryland, Massachusetts, Michigan, Minnesota, Mississippi, Missouri, Nevada, New Hampshire, New Jersey, New York, New York City, North Carolina, Ohio, Oklahoma, Oregon, Pennsylvania, Rhode Island, Texas, Vermont, Virginia, Washington, West Virginia, and Wisconsin). For 2010, a total of 37 jurisdictions reported data to CDC (Alabama, Arizona, California, Connecticut, Delaware, District of Columbia, Florida, Georgia, Illinois, Indiana, Iowa, Kansas, Kentucky, Louisiana, Maine, Maryland, Massachusetts, Michigan, Minnesota, Mississippi, Missouri, Nevada, New Hampshire, New Jersey, New York, New York City, Ohio, Oklahoma, Oregon, Pennsylvania, Rhode Island, Texas, Vermont, Virginia, Washington, West Virginia, and Wisconsin). For 2011, a total of 36 jurisdictions reported data to CDC (Alabama, Arizona, California, Connecticut, Delaware, District of Columbia, Florida, Georgia, Illinois, Indiana, Iowa, Kansas, Kentucky, Louisiana, Maine, Maryland, Massachusetts, Michigan, Minnesota, Mississippi, Missouri, New Hampshire, New Jersey, New York, New York City, Ohio, Oklahoma, Oregon, Pennsylvania, Rhode Island, Texas, Vermont, Virginia, Washington, West Virginia, and Wisconsin). For 2012, a total of 30 jurisdictions reported data to CDC (Alabama, Arizona, Connecticut, District of Columbia, Florida, Georgia, Illinois, Indiana, Iowa, Kansas, Kentucky, Louisiana, Maryland, Massachusetts, Michigan, Minnesota, Mississippi, Missouri, New Hampshire, New Jersey, New York, New York City, Ohio, Oklahoma, Oregon, Pennsylvania, Rhode Island, Vermont, West Virginia, and Wisconsin). For 2013, a total of 29 jurisdictions reported data to CDC (Alabama, Arizona, Connecticut, District of Columbia, Georgia, Illinois, Indiana, Kentucky, Louisiana, Maryland, Massachusetts, Michigan, Minnesota, Mississippi, Missouri, New Hampshire, New Jersey, New Mexico, New York City, North Carolina, Ohio, Oklahoma, Oregon, Pennsylvania, Rhode Island, Tennessee, Vermont, West Virginia, and Wisconsin). For 2014, a total of 32 jurisdictions reported data to CDC (Alabama, Arizona, Colorado, Connecticut, District of Columba, Georgia, Illinois, Indiana, Kentucky, Louisiana, Maryland, Massachusetts, Michigan, Minnesota, Mississippi, Missouri, New Hampshire, New Jersey, New Mexico, New York, New York City, North Carolina, Ohio, Oklahoma, Oregon, Pennsylvania, Rhode Island, Tennessee, Texas, Vermont, West Virginia, and Wisconsin).

**FIGURE 2:**
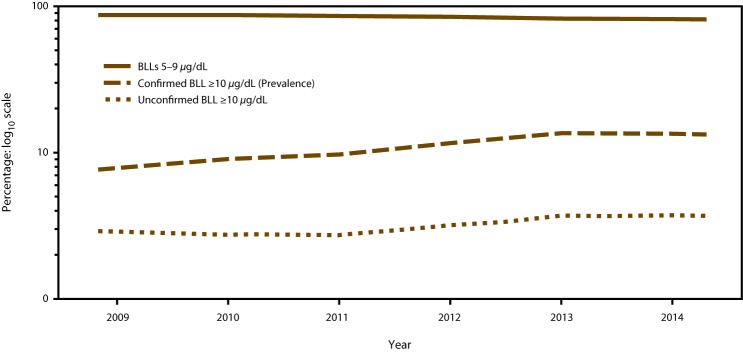
Percentage of children aged <5 years with blood lead levels ≥5 *μ*g/dL, by year and blood lead level — Childhood Blood Lead Surveillance System, United States, 2009–2014* **Abbreviation:** BLLs = blood lead levels. *For 2009, a total of 38 jurisdictions reported data to CDC (Alabama, Arizona, California, Connecticut, Delaware, District of Columbia, Florida, Georgia, Illinois, Indiana, Iowa, Kansas, Kentucky, Louisiana, Maine, Maryland, Massachusetts, Michigan, Minnesota, Mississippi, Missouri, Nevada, New Hampshire, New Jersey, New York, New York City, North Carolina, Ohio, Oklahoma, Oregon, Pennsylvania, Rhode Island, Texas, Vermont, Virginia, Washington, West Virginia, and Wisconsin). For 2010, a total of 37 jurisdictions reported data to CDC (Alabama, Arizona, California, Connecticut, Delaware, District of Columbia, Florida, Georgia, Illinois, Indiana, Iowa, Kansas, Kentucky, Louisiana, Maine, Maryland, Massachusetts, Michigan, Minnesota, Mississippi, Missouri, Nevada, New Hampshire, New Jersey, New York, New York City, Ohio, Oklahoma, Oregon, Pennsylvania, Rhode Island, Texas, Vermont, Virginia, Washington, West Virginia, and Wisconsin). For 2011, a total of 36 jurisdictions reported data to CDC (Alabama, Arizona, California, Connecticut, Delaware, District of Columbia, Florida, Georgia, Illinois, Indiana, Iowa, Kansas, Kentucky, Louisiana, Maine, Maryland, Massachusetts, Michigan, Minnesota, Mississippi, Missouri, New Hampshire, New Jersey, New York, New York City, Ohio, Oklahoma, Oregon, Pennsylvania, Rhode Island, Texas, Vermont, Virginia, Washington, West Virginia, and Wisconsin). For 2012, a total of 30 jurisdictions reported data to CDC (Alabama, Arizona, Connecticut, District of Columbia, Florida, Georgia, Illinois, Indiana, Iowa, Kansas, Kentucky, Louisiana, Maryland, Massachusetts, Michigan, Minnesota, Mississippi, Missouri, New Hampshire, New Jersey, New York, New York City, Ohio, Oklahoma, Oregon, Pennsylvania, Rhode Island, Vermont, West Virginia, and Wisconsin). For 2013, a total of 29 jurisdictions reported data to CDC (Alabama, Arizona, Connecticut, District of Columbia, Georgia, Illinois, Indiana, Kentucky, Louisiana, Maryland, Massachusetts, Michigan, Minnesota, Mississippi, Missouri, New Hampshire, New Jersey, New Mexico, New York City, North Carolina, Ohio, Oklahoma, Oregon, Pennsylvania, Rhode Island, Tennessee, Vermont, West Virginia, and Wisconsin). For 2014, a total of 32 jurisdictions reported data to CDC (Alabama, Arizona, Colorado, Connecticut, District of Columba, Georgia, Illinois, Indiana, Kentucky, Louisiana, Maryland, Massachusetts, Michigan, Minnesota, Mississippi, Missouri, New Hampshire, New Jersey, New Mexico, New York, New York City, North Carolina, Ohio, Oklahoma, Oregon, Pennsylvania, Rhode Island, Tennessee, Texas, Vermont, West Virginia, and Wisconsin).

This report provides data concerning childhood blood lead levels (BLLs) in the United States during 2009–2014. These data were collected and compiled from raw data extracts sent by state and local health departments to CDC’s Childhood Blood Lead Surveillance (CBLS) system. These raw data extracts have been de-identified and coded into a format specifically for childhood blood lead reporting. The numbers of children aged <5 years for 2014 are reported with newly confirmed BLLs ≥10 *µ*g/dL by month (Table 1) and geographic location (Table 2). The incidence of BLLs ≥10 *µ*g/dL is reported by age group for 2009–2014 (Table 3). The numbers of children aged <5 years are reported by the prevalence of BLLs 5–9 *µ*g/dL by age group and sample type during 2009–2014 (Tables 4 and 5). For the period 2009–2014, the numbers of children newly confirmed with BLLs ≥70 *µ*g/dL are summarized (Figure 1) as well as the percentage of children with BLLs ≥5 *µ*g/dL (Figure 2).

**TABLE 1:** Number and percentage of reported new cases of blood lead levels ≥10 *µ*g/dL among children aged <5 years, by month — Childhood Blood Lead Surveillance System, United States, 2014*

Jan	Feb	Mar	Apr	May	Jun	Jul	Aug	Sept	Oct	Nov	Dec	Total No.
No. (%)	No. (%)	No. (%)	No. (%)	No. (%)	No. (%)	No. (%)	No. (%)	No. (%)	No. (%)	No. (%)	No. (%)	
473 (5.3)	420 (4.7)	510 (5.8)	551 (6.2)	626 (7.1)	819 (9.3)	966 (10.9)	1,008 (11.4)	1,129 (12.8)	1,053 (11.9)	717 (8.1)	584 (6.6)	**8,856**

*A total of 32 jurisdictions reported data to CDC (Alabama, Arizona, Colorado, Connecticut, District of Columba, Georgia, Illinois, Indiana, Kentucky, Louisiana, Maryland, Massachusetts, Michigan, Minnesota, Mississippi, Missouri, New Hampshire, New Jersey, New Mexico, New York, New York City, North Carolina, Ohio, Oklahoma, Oregon, Pennsylvania, Rhode Island, Tennessee, Texas, Vermont, West Virginia, and Wisconsin).

**TABLE 2:** Reported number of newly identified cases of blood lead levels ≥10 *µ*g/dL among children aged <5 years, by geographic region and state — Childhood Blood Lead Surveillance System, United States, 2014

Region/State	No.
**United States**	**8,856**	
**New England**	**1,152**	
Connecticut	338	
Maine	—*	
Massachusetts	563	
New Hampshire	59	
Rhode Island	156	
Vermont	36	
**Mid Atlantic**	**2,865**	
New Jersey	565	
New York	516	
New York City	677	
Pennsylvania	1,107	
**Eastern North Central**	**3,157**
Illinois	1,061	
Indiana	151	
Michigan	485	
Ohio	1,030	
Wisconsin	430	
**Western North Central**	**626**
Iowa	—	
Kansas	—	
Minnesota	173	
Missouri	453	
Nebraska	—	
North Dakota	—	
South Dakota	—	
**South Atlantic**	**492**	
Delaware	—	
District of Columbia	40	
Florida	—	
Georgia	100	
Maryland	227	
North Carolina	113	
South Carolina	—	
Virginia	—	
West Virginia	12	
**Eastern South Central**	**243**	
Alabama	87	
Kentucky	38	
Mississippi	57	
Tennessee	61	
**Western South Central**	**231**	
Arkansas	—	
Louisiana	81	
Oklahoma	148	
Texas	2	
**Mountain**	**90**	
Arizona	51	
Colorado	25	
Idaho	—	
Montana	—	
Nevada	—	
New Mexico	14	
Utah	—	
Wyoming	—	
**Pacific**	**0**	
Alaska	—	
California	—	
Hawaii	—	
Oregon	0	
Washington	—	

*No data were reported for 2014.

**TABLE 3:** Reported number of new cases and incidence rate per 100,000 children aged <5 years with blood lead levels ≥10 *µ*g/dL, by age group — Childhood Blood Lead Surveillance System, United States, 2009–2014*

Year	<1 yr		1–4 yrs	
	No.	Rate	No.	Rate
2009^†^	1,608	38.69	3,432	78.76
2010^§^	1,412	34.05	11,647	68.05
2011^¶^	1,185	29.89	10,532	65.25
2012**	860	21.81	9,369	58.31
2013^††^	777	19.55	7,453	46.89
2014^§§^	791	19.90	8,056	50.66

*Denominators are calculated as estimates of all children living in the United States from U.S. Census data for 2009–2014.

^†^A total of 38 jurisdictions reported data to CDC (Alabama, Arizona, California, Connecticut, Delaware, District of Columbia, Florida, Georgia, Illinois, Indiana, Iowa, Kansas, Kentucky, Louisiana, Maine, Maryland, Massachusetts, Michigan, Minnesota, Mississippi, Missouri, Nevada, New Hampshire, New Jersey, New York, New York City, North Carolina, Ohio, Oklahoma, Oregon, Pennsylvania, Rhode Island, Texas, Vermont, Virginia, Washington, West Virginia, and Wisconsin).

^§^A total of 37 jurisdictions reported data to CDC (Alabama, Arizona, California, Connecticut, Delaware, District of Columbia, Florida, Georgia, Illinois, Indiana, Iowa, Kansas, Kentucky, Louisiana, Maine, Maryland, Massachusetts, Michigan, Minnesota, Mississippi, Missouri, Nevada, New Hampshire, New Jersey, New York, New York City, Ohio, Oklahoma, Oregon, Pennsylvania, Rhode Island, Texas, Vermont, Virginia, Washington, West Virginia, and Wisconsin).

^¶^A total of 36 jurisdictions reported data to CDC (Alabama, Arizona, California, Connecticut, Delaware, District of Columbia, Florida, Georgia, Illinois, Indiana, Iowa, Kansas, Kentucky, Louisiana, Maine, Maryland, Massachusetts, Michigan, Minnesota, Mississippi, Missouri, New Hampshire, New Jersey, New York, New York City, Ohio, Oklahoma, Oregon, Pennsylvania, Rhode Island, Texas, Vermont, Virginia, Washington, West Virginia, and Wisconsin).

**A total of 30 jurisdictions reported data to CDC (Alabama, Arizona, Connecticut, District of Columbia, Florida, Georgia, Illinois, Indiana, Iowa, Kansas, Kentucky, Louisiana, Maryland, Massachusetts, Michigan, Minnesota, Mississippi, Missouri, New Hampshire, New Jersey, New York, New York City, Ohio, Oklahoma, Oregon, Pennsylvania, Rhode Island, Vermont, West Virginia, and Wisconsin).

^††^A total of 29 jurisdictions reported data to CDC (Alabama, Arizona, Connecticut, District of Columbia, Georgia, Illinois, Indiana, Kentucky, Louisiana, Maryland, Massachusetts, Michigan, Minnesota, Mississippi, Missouri, New Hampshire, New Jersey, New Mexico, New York City, North Carolina, Ohio, Oklahoma, Oregon, Pennsylvania, Rhode Island, Tennessee, Vermont, West Virginia, and Wisconsin).

^§§^ A total of 32 jurisdictions reported data to CDC (Alabama, Arizona, Colorado, Connecticut, District of Columba, Georgia, Illinois, Indiana, Kentucky, Louisiana, Maryland, Massachusetts, Michigan, Minnesota, Mississippi, Missouri, New Hampshire, New Jersey, New Mexico, New York, New York City, North Carolina, Ohio, Oklahoma, Oregon, Pennsylvania, Rhode Island, Tennessee, Texas, Vermont, West Virginia, and Wisconsin).

**TABLE 4:** Number and rate per 100,000 children aged <5 years with blood lead levels 5–9 *µ*g/dL, by age group — Childhood Blood Lead Surveillance System, United States, 2010–2014*

Year	<1 yr		1–4 yrs	
	No.	Rate	No.	Rate
2010^†^	18,598	448.48	137,887	805.62
2011^§^	13,981	352.69	130,838	810.56
2012^¶^	7,876	199.74	95,854	596.58
2013**	5,494	138.26	57,293	360.46
2014^††^	5,904	148.51	70,680	444.49

*Denominators are calculated as estimates of all children living in the United States from U.S. Census data for 2009–2014.

^†^A total of 37 jurisdictions reported data to CDC (Alabama, Arizona, California, Connecticut, Delaware, District of Columbia, Florida, Georgia, Illinois, Indiana, Iowa, Kansas, Kentucky, Louisiana, Maine, Maryland, Massachusetts, Michigan, Minnesota, Mississippi, Missouri, Nevada, New Hampshire, New Jersey, New York, New York City, Ohio, Oklahoma, Oregon, Pennsylvania, Rhode Island, Texas, Vermont, Virginia, Washington, West Virginia, and Wisconsin).

^§^A total of 36 jurisdictions reported data to CDC (Alabama, Arizona, California, Connecticut, Delaware, District of Columbia, Florida, Georgia, Illinois, Indiana, Iowa, Kansas, Kentucky, Louisiana, Maine, Maryland, Massachusetts, Michigan, Minnesota, Mississippi, Missouri, New Hampshire, New Jersey, New York, New York City, Ohio, Oklahoma, Oregon, Pennsylvania, Rhode Island, Texas, Vermont, Virginia, Washington, West Virginia, and Wisconsin).

^¶^A total of 30 jurisdictions reported data to CDC (Alabama, Arizona, Connecticut, District of Columbia, Florida, Georgia, Illinois, Indiana, Iowa, Kansas, Kentucky, Louisiana, Maryland, Massachusetts, Michigan, Minnesota, Mississippi, Missouri, New Hampshire, New Jersey, New York, New York City, Ohio, Oklahoma, Oregon, Pennsylvania, Rhode Island, Vermont, West Virginia, and Wisconsin).

**A total of 29 jurisdictions reported data to CDC (Alabama, Arizona, Connecticut, District of Columbia, Georgia, Illinois, Indiana, Kentucky, Louisiana, Maryland, Massachusetts, Michigan, Minnesota, Mississippi, Missouri, New Hampshire, New Jersey, New Mexico, New York City, North Carolina, Ohio, Oklahoma, Oregon, Pennsylvania, Rhode Island, Tennessee, Vermont, West Virginia, and Wisconsin).

^††^A total of 32 jurisdictions reported data to CDC (Alabama, Arizona, Colorado, Connecticut, District of Columba, Georgia, Illinois, Indiana, Kentucky, Louisiana, Maryland, Massachusetts, Michigan, Minnesota, Mississippi, Missouri, New Hampshire, New Jersey, New Mexico, New York, New York City, North Carolina, Ohio, Oklahoma, Oregon, Pennsylvania, Rhode Island, Tennessee, Texas, Vermont, West Virginia, and Wisconsin).

**TABLE 5:** Number of children aged <5 years with blood lead levels 5–9 *µ*g/dL, by sample type and age — Childhood Blood Lead Surveillance System, United States, 2014*

Age at time of blood lead test (yrs)	Capillary/Unknown (%)	Venous (%)
<1	3,681 (62.4)	2,223 (37.7)
1	13,997 (57.5)	10,334 (42.5)
2	11,992 (59.4)	8,190 (40.6)
3	6,624 (60.0)	4,425 (40.0)
4	5,418 (58.8)	3,796 (41.2)
**Total**	**41,712 (59.0)**	**28,968 (41.0)**

*A total of 32 jurisdictions reported data to CDC (Alabama, Arizona, Colorado, Connecticut, District of Columba, Georgia, Illinois, Indiana, Kentucky, Louisiana, Maryland, Massachusetts, Michigan, Minnesota, Mississippi, Missouri, New Hampshire, New Jersey, New Mexico, New York, New York City, North Carolina, Ohio, Oklahoma, Oregon, Pennsylvania, Rhode Island, Tennessee, Texas, Vermont, West Virginia, and Wisconsin).

## Background

No safe BLLs in children have been identified (*1*). Permanent neurologic damage and behavioral disorders are associated with BLLs at or below 5 *µ*g/dL (*2*–*5*). Studies examining children with high BLLs (≥70 *µ*g/dL) describe severe neurologic problems, including seizures, comas, and death (*6*). Children aged <5 years are at increased risk because their bodies are growing rapidly and they tend to put their hands or other objects, which might be contaminated with lead dust, into their mouths.

In 1991, CDC recommended that BLLs ≥10 *µ*g/dL in children should prompt public health action by the state or local health departments with follow-up testing (*7*). In 1995, the Council of State and Territorial Epidemiologists (CSTE), in collaboration with CDC, designated elevated BLLs as the first noninfectious condition to be added to the list of conditions designated as reportable at the national level (*8*).

In May 2012, the Advisory Committee on Childhood Lead Poisoning Prevention* (ACCLPP) recommended the use of a reference range to identify elevated BLLs (*7*). ACCLPP recommended that the upper value of the reference range be based on the 97.5th percentile of the National Health and Nutritional Examination Survey^†^ (NHANES). The current value (5 *µ*g/dL) is used by clinical and public health care providers to identify children with elevated BLLs (*9*).

In 2012, a total of 30 jurisdictions (28 states, the District of Columbia, and New York City) identified and reported approximately 138,000 children aged <6 years with BLLs ≥5 *µ*g/dL (*10*). Federal funding ended in 2012, and several states lost their state-wide lead programs. As a result, by 2013, the number of children reported to CDC with BLLs ≥5*µ*g/dL decreased, as did the number of states reporting (*10*). When federal funding resumed in October 2013, a total of 35 programs, including 29 state health departments, the District of Columbia, and five local programs, reported data to CDC. For this report, CDC examined BLLs of children aged <5 years in three categories: children with BLLs ≥10 *µ*g/dL, children with new reports of BLLs ≥10 *µ*g/dL, and children with BLLs 5–9 *µ*g/dL.

## Data Sources

Results of all blood lead tests for children in this surveillance summary were sent to CDC’s Healthy Homes and Lead Poisoning Prevention Program (HHLPPP). When federal funding was available, from the early 1990s–2012, states submitted data on a quarterly basis. After funding ended, 27 states, the District of Columbia and New York City continued to submit data to CDC while the other states lost their childhood lead programs and could not provide data. For the states that continued to have childhood lead programs, most had sustainable funding through the state where the health departments would verify the data collected for blood lead testing. When federal funding returned in 2014, a total of 35 programs were funded through CDC.^§^ Test results compiled and de-identified by state and local health departments and submitted to CDC comprise the CBLS database.

State and local childhood blood lead surveillance systems retain the results of blood lead tests of children reported to state health departments by private laboratories, as well as state and local government laboratories. The reporting criteria of BLLs from the laboratories to the state are set by each state and vary across jurisdictions. A set of core data variables have been established by CDC and participating states that should be collected for every child at the time of the blood lead test. These variables include identification and demographic information (e.g., date of birth, race, or ethnicity), laboratory information (e.g., venous or capillary blood test), date of blood lead test, address information such as city and zip code, and test result (*11*). Each child is assigned a unique identifier that corresponds to the de-identified and de-duplicated data sent to CDC along with the core data. CDC checks each state-submitted record for correct formatting, coding and content. Records not meeting CDC criteria are summarized in file processing reports that are sent to states for correction. Certain errors, if not corrected, prevent the record from being entered in CDC’s CBLS database. For states with an error rate >10% rate, no data are loaded into CBLS, and the state must correct the problems before sending the records back to CDC.

## Interpreting Data

In this report, state surveillance data are presented for children aged <5 years who were tested for lead at least once during 2009–2014 and whose tests were reported to CDC. Having a confirmed BLL ≥10 *µ*g/dL is defined as having one venous blood lead test ≥10 *µ*g/dL or two capillary blood lead tests ≥10 *µ*g/dL drawn within 12 weeks of each other (*12*). Incidence data rates are presented by the date of the confirmed blood lead test. Data are reported by the jurisdiction of the child’s residence at the time of the confirmed blood lead test. State and local health departments check for duplicate laboratory reports for children and completeness of the laboratory report before sending the data extracts to CDC. After data are sent, CDC also checks for completeness and accuracy of the data.

The data provided in this report are useful for analyzing childhood blood lead trends and determining relative morbidity numbers. However, reporting practices affect how these data are interpreted. Childhood blood lead reporting is likely incomplete, and completeness of the records might vary depending on state, laboratory, or BLL range (e.g., BLLs <10 *µ*g/dL might not be required to be reported in some states). Independent of the actual incidence of disease, factors such as changes in the methods of surveillance or introduction of new diagnostic tests (e.g., portable handheld analyzer) can cause changes in the reported blood lead levels. With these limitations of data collection methods varying across states, data cannot be compared across states or counties because data collection methods vary across grantees.

In 2009, a total of 36 states, the District of Columbia, and New York City reported data to CDC. During 2009–2014, the number of states collecting and reporting childhood blood lead data to CDC fluctuated. Federal funding from CDC to state and local health departments ended in September 2012. For this reason, no states were required to report childhood blood lead data to CDC in 2013. Nevertheless, 29 jurisdictions (27 states, the District of Columbia, and New York City) did collect and report data to CDC. Although the varying number of states reporting data from year to year limits the extent of trend analyses that can be done, the data nonetheless can indicate BLLs in children for a particular year. At the end of 2013, federal funding resumed providing cooperative agreements to 29 states, the District of Columbia, and five local programs.

## Methods for Identifying Elevated Childhood Blood Lead Levels

Elevated BLLs have been a notifiable condition since 1995 (*7*). CDC asks state and local health departments to report all blood lead test data for children to HHLPPP, regardless of the BLL. Each state has its own laws and regulations regarding blood lead test reporting to the state health department. Of the 35 programs funded in 2014 by CDC, 33 required electronic reporting from the laboratory to the state health department.

Before ACCLPP’s recommendation in May 2012, the level for which public health intervention was warranted for children aged <6 years was ≥10 *µ*g/dL (*8*). Since then, the reference level has shifted to ≥5 *µ*g/dL (*9*). For this report, elevated blood lead levels are defined as confirmed BLLs ≥10 *µ*g/dL. Data on children with BLLs ≥5 *µ*g/dL also are reported because the change to a reference value did not occur until mid-2012 and federal funding from CDC to state and local health departments ended in September 2012 and resumed in late 2014.

## Publication Criteria

Reports of children aged <5 years with BLLs 5–9 *µ*g/dL and confirmed BLLs ≥10 *µ*g/dL during 2009–2014.

## Highlights

Lead exposure in children can cause permanent neurological damage (*1*). Behavioral disorders are associated with lead exposure even at detectable blood levels at or below 5 *µ*g/dL (*1*–*4*). The most common highly concentrated source of lead for children in the United States is lead paint. When paint containing lead deteriorates into flakes, chips, or fine dust, it is easily inhaled or ingested by small children.

In 2011, a total of 34 states, the District of Columbia, and New York City submitted BLL data to CDC; however by 2013, only 27 states, the District of Columbia, and New York City submitted data (a 17% reduction in contributors). By 2014, the number of states reporting childhood blood lead data increased to 30 states, the District of Columbia, and New York City. Although the decrease in state and local health departments submitting data to CDC makes it difficult to assess the trend over time, it is still possible to evaluate new cases of children with confirmed BLLs ≥10 *µ*g/dL and cases in children with BLLs 5–9 *µ*g/dL from the jurisdictions that continue to submit data to CDC.

In 2014, during the warmest weather months (August–October), 36% of new cases were identified, more than any other consecutive 3-month period (Table 1). In warm weather, windows possibly painted with lead-based paint are opened and closed, creating lead dust in the air and on the ground (*13*). Repainting and renovation activities also are more common in warmer months. Increased presence and activity of children in and around the home might lead to children having more contact with contaminated dust, surfaces, and soil (*14*). This contact can lead to higher BLLs in the late summer and early fall.

The East North Central region reported the greatest number of new cases in 2014 with 3,157 children aged <5 years with newly confirmed BLLs ≥10 *µ*g/dL reported to CDC, followed by the Mid-Atlantic region, with 2,865 children (Table 2). These two regions (comprising nine state and local health departments that reported data) accounted for 68% of the new cases in the United States and for approximately 49% of the children aged <5 years tested and reported to CDC for 2014 (data not shown). The other seven geographic areas (comprising 23 jurisdictions that reported data to CDC for 2014) accounted for the remaining 32% of new cases and for 51% of the children aged <5 years tested and reported to CDC for 2014.

The number of children aged <5 years with a first-ever confirmed BLL ≥10 *µ*g/dL reported to CDC, continued to decrease from 2009 to 2013, but increased in 2014 when federal funding was restored (Table 3). Although not all jurisdictions reported data to CDC, the denominator is the entire child population aged <1 year and aged 1–4 years from the U.S. Census across all years. Children aged 1–4 years continue to have a higher rate of confirmed BLLs ≥10 *µ*g/dL than children <1 year across all years (50.7% vs 19.9% respectively), possibly because of increased hand-to-mouth activity and mobility for younger children. The prevalence of children aged <5 years with a BLL 5–9 *µ*g/dL also declined until 2014 (Table 4). The rate of decline for children with BLLs 5–9 *µ*g/dL (Table 4) is sharper than the rate for children with newly confirmed BLLs ≥10 µg/dL (Table 3) over time (67% and 45% versus 49% and 36%).

The numbers of newly confirmed children with BLLs ≥70 *µ*g/dL remains inconsistent over the past 6 years. Changes in the number of states reporting data to CDC over these 6 years make it difficult to show any trend or clear pattern. One *Healthy People 2010* environmental health objective was eliminating BLLs ≥10 *µ*g/dL (*15*). These children have BLL’s at least seven times above the *Healthy People 2010* goal.

Prevalence data indicate that 76,680 children aged <5 years had BLLs 5–9 *µ*g/dL in 2014 (Table 5). A blood lead test is collected through either a capillary or venous sample. In a small number of tests, the sample type was unknown, and for analysis purposes, was combined with capillary samples. A single capillary test is not a confirmatory test, but in 2014, of the 76,680 children aged <5 years tested and reported to CDC with BLLs 5–9 *µ*g/dL, 41% had a confirmed BLL 5–9 *µ*g/dL by venous sample type. The change in the reference value in 2012, and the loss of federal funding to state and local health departments from CDC, made it difficult for most states to extend follow up testing for capillary tests 5–9 *µ*g/dL. Although venous blood lead samples have been the gold standard, one study has shown that capillary blood draws are suitable alternatives to venous blood draws when screening children aged <6 years to determine lead exposure and provide reasonable estimates at the population level (*16*).

During 2009–2011, a majority of the children aged <5 years with BLLs ≥5 *µ*g/dL had BLLs 5–9 *µ*g/dL (Figure 2). The percentage of children with confirmed BLLs ≥10 *µ*g/dL increased from 7.6% to 13.4% during the same period. CDC, along with state and local health departments, continues efforts to reduce BLLs ≥5 *µ*g/dL and confirmed BLL ≥10 *µ*g/dL through screening and primary prevention (*17*).

Effective surveillance requires state and local health departments to track a substantial number of children and their blood lead test results over time. More detailed annual summaries describing the number of children tested for lead by state, county, and BLL are published periodically by CDC; a summary of childhood lead exposure in 2014, the most recent year for which data is available, is provided at https://www.cdc.gov/nceh/lead.

## Footnotes

* ACCLPP advised and guided the Secretary and Assistant Secretary of the U.S. Department of Health and Human Services and the Director of CDC regarding new scientific knowledge and technical developments and their practical implications for childhood lead poisoning prevention efforts (https://www.cdc.gov/nceh/lead/acclpp/acclpp_main.htm).

† NHANES is a program of studies designed to assess the health and nutritional status of adults and children in the United States (https://www.cdc.gov/nchs/nhanes.htm).

§ Of the 35 jurisdictions funded in 2014, a total of 32 jurisdictions reported data to CDC (Alabama, Arizona, Colorado, Connecticut, District of Columba, Georgia, Illinois, Indiana, Kentucky, Louisiana, Maryland, Massachusetts, Michigan, Minnesota, Mississippi, Missouri, New Hampshire, New Jersey, New Mexico, New York, New York City, North Carolina, Ohio, Oklahoma, Oregon, Pennsylvania, Rhode Island, Tennessee, Texas, Vermont, West Virginia, and Wisconsin). Three jurisdictions did not report data to CDC.
